# Decision Making in Health Management during Crisis: A Case Study Based on Epidemiological Curves of China and Italy against COVID-19

**DOI:** 10.3390/ijerph18158078

**Published:** 2021-07-30

**Authors:** Salvador Ávila Filho, Júlia Spínola Ávila, Beata Mrugalska, Naiara Fonseca de Souza, Ana Paula Meira Gomes de Carvalho, Lhaís Rodrigues Gonçalves

**Affiliations:** 1 Department of Mechanical Engineering, Polytechnic School, Federal University of Bahia, Salvador 40210-630, Brazil; avilasalva@gmail.com; 2 Institute of Humanities, Arts and Sciences Professor Milton Santos, Federal University of Bahia, Salvador 40170-115, Brazil; julia.savilaa@gmail.com (J.S.Á.); fonsecanaiara0@gmail.com (N.F.d.S.); anapaulameiragcarvalho@gmail.com (A.P.M.G.d.C.); lhaisrodrigues2@outlook.com (L.R.G.); 3 Faculty of Engineering Management, Poznan University of Technology, 60-965 Poznan, Poland

**Keywords:** COVID-19, China, Italy, parameters, health management, crisis management

## Abstract

In December 2019, a new infectious respiratory disease called COVID-19 was identified in Wuhan, Hubei province, in China and quickly reached pandemic status in March 2020, in uncertain and frightening situation. The objective of this study was to analyze the epidemiological curves from the fight against COVID-19 in China and Italy, establishing parameters that can assist with the decisions of health-planning managers. This study was conducted using the principles of the grounded theory methodology and a practical method of comparison between the real and ideal curves, based on the contamination and death data by SARS-CoV-2 in China and Italy. For this purpose, we built graphs, including parameters, such as, among others, amplitude, height, saturation point, acceleration, lethality, event, risk, and efficiency. The results of our study showed that China exhibited amplitude and height of the active contamination and death curve 2 times smaller than those of Italy which exhibited several saturations. It was investigated that Italy presented a qualitative risk of 5–6, whereas for China it was 4. According to the parameters, China and Italy presented health management that was able to reduce the impact caused by the virus. The implementation of adequate health management with these practical tools can guide perception of the crisis critical levels, avoiding major disasters. We intend to continue to validate the method in the analysis of data from Brazil and the USA.

## 1. Introduction

The first case records of COVID-19 in the world appeared in December 2019, in Wuhan, China and led to a global pandemic in March 2020 [1]. As it appeared in an unexpected, isolated way with high global impacts and low predictability [2], this phenomenon could be related to the event called “Black Swan”, proposed by Taleb [3]. It was a noticeable scenario of unpreparedness regarding the perception of the signs of COVID-19 as the hazard energy passed without the installation of barriers in any sector, promoting a chain reaction until reaching the “great damage” [4]. Therefore, it was necessary to establish integrated crisis management, which could enable the integration of the collection and monitoring of deaths and contaminated indicators associated with corrective actions. Such actions could help to contain failures, resulting from the lack of knowledge about the hazard, cultural biases and the lack of leadership [5]. The urgency of elaborating the safeguards was due to the non-resolution of the pandemic and the need to determine assertive measures [6].

Integrated crisis management must include skills from different areas of expertise and knowledge, constituting a complex phenomenon to plan and implement actions. The various disciplines involved in the treatment of this phenomenon are demanded with a certain humility and a good level of organization to make appropriate decisions to reduce the impacts of COVID-19 in different dimensions [7]. It is essential to know the technical and socio-demographic nature of the COVID-19 phenomenon for the organization of disordered data, resulting from the uncertainties that are involved in a lack of knowledge [8]. The intention is to know where and when to install the barriers that will contain the hazard energy to avoid effects such as high number of dead and contaminated people and, in the long run, poverty, unemployment and hunger.

The analysis of resource constraints and the search for the flattening of the epidemiological curve [9] during the crisis can project different strategies, resulting in the respective scenarios. In this paper, the authors proposed three different scenarios. In Scenario 1, the reduction in the height of the contamination curve was represented through social isolation and/or quarantine, softening the impacts and preserving the health system. Scenario 2, intermediate, arose from the lack of knowledge linked to the lack of resources or the fear of crashing the country’s economy. In this case, there was a new wave of contamination in the initial curve with an increase in the contamination rate due to an early removal of the restriction measures or lack of investment in the right amount and time. This situation resulted in a rework of social isolation and restrictions postponing the end of the event. Scenario 3 was the result of the government’s inadequate acknowledgement who did not treat COVID-19 with the necessary responsibility by delegitimizing its power to generate damage, creating a generalized disorder without social isolation.

In the elaboration of the tools for the decision on the strategies, the initial cases of “success” in health management were studied, as they had great similarity with the model considered to be an ideal curve referring to half of a sine cycle. This study adhered to elements contained in the Grounded Theory methodology in which the theory was developed in an inductive, interactive, and simultaneous way, grouping, evaluating and categorizing the data [10].

In the paper, we studied the cases from China and Italy, classified as Scenario 1 with differences in impacts and approximation to the ideal model. According to the collected data, Italy was a case study that visually ended the first cycle, practically zeroing the impacts, but which suffered again with a new wave, which will not be studied in this paper.

China was the first country to face COVID-19, Hubei being the most affected province. Facing the need to curb the spread of the virus, the Chinese government, after just three weeks from the beginning of the epidemic, adopted more severe measures. It was based on community confinement in the city of Wuhan, the epicenter of COVID-19, and in the quarantine of 15 more cities in the Hubei province the next day. Ensuring strict compliance with quarantine measures, despite all efforts, there was a significant increase in the cases and the spread of SARS-CoV-2 in February, although it did not reach the entire country [11,12,13,14]. Considering the incipient discoveries of the contamination and propagation of the new coronavirus, the quarantine began with rigor, through travel restrictions, closing public places, suspension of classes and labor services (except essential activities), and the cancellation of events, besides the limitation of any external activity [14]. However, China employed enough resources to control the spread of the epidemic and strengthen its health system, highlighting the expansion of testing and access to the treatment through government resources. In addition, this country provided a high speed in the execution of activities, such as the construction of hospitals, production of inputs, and support to the population [14]. The gradual resumption of socio-economic activities included the practice of disinfecting all locations, mandatory use of a mask, temperature measurement, 1.5 m distancing, testing for early detection and the creation of new technologies to ensure contact tracking and the monitoring of people in the risk group [14]. The discipline of this eastern country with a communist regime indicated one of the main characteristics for the success of the work with regard to the quarantine rules and efficiency in fighting the virus: committing to the restrictive measures [15]. In short, this reality contributed to the similarity of this country’s epidemiological curve to that presented in Scenario 1.

Italy was the first European country affected by the new coronavirus and became the world epicenter in the terms of the number of cases and deaths in March. The epidemic spread mainly into the northern region of the country [16], where people over 80 and health professionals, who worked on the front line against the coronavirus, corresponded to the most contaminated population and comprised a great number of the fatalities [17]. This situation occurred due to the low availability of personal protective equipment (PPE) in the initial stages of the pandemic [8] and overcrowding in hospitals, creating a sequence that contributed to the rapid spread of the virus in the country [16].

According to Torri et al. [18], on 21 February 2020, the first death by COVID-19 was documented, motivating the public governance to adopt more measures against the epidemic, despite neglecting the seriousness of emerging health issues and prioritizing the economic sector. Among the adopted measures, there was the use of an app tool for voluntary people tracking in Lombardy [8], the establishment of government information system, the strengthening of local health services, continuous supply of PPE, kits for laboratory analysis and devices for respiratory support [19], which were extremely important, especially in the period between March and April, a time of crisis for the country. Since April, a response to public measures was made [18]. In line with FNOMCeO [19], on 4 May 2020, a phase of lockdown flexibilization was initiated in Italy. There was a reopening of trade and non-essential activities, guaranteeing a return to the stability of the economy, but with responsibility through personal care. Despite the initial lack of control of the pandemic in the country, the actions were taken effectively and presented satisfactory results in the fight against the coronavirus, bringing the country’s trajectory closer to that presented in Scenario 1.

As public health management seeks to develop situations to meet the health needs of the population, it is important to assess its efficiency on the basis of the impacts, indicating the most effective control measures to reduce the impact of the epidemic curve by reducing contagion. From this perspective, the objective of this study was the analysis of the epidemiological curve based on the containment of COVID-19 in China and Italy, establishing parameters to assist in the decision of health planning managers. In the face of new crises, especially pandemics, these indicators can guide the period and the intensity in which restrictive measures must be applied.

## 2. Materials and Methods

The paper shows a qualitative–quantitative, exploratory study based on the principles of Grounded Theory (GT) as a guiding methodology in the collection and analysis of data on the contaminated and deaths by SARS-CoV-2 in China and Italy. Regarding GT, the objective was to find latent patterns among any type of data to develop general theories in an inductive way [20], configuring a “grounded” theory in real data, in which hypotheses, arising from current paradigms and theories, should not be previously formed [21,22].

As it is classified as an exploratory methodology, GT stands out in themes that need greater depth or breadth and that are extensively dynamic, such as the unexpected phenomenon of the COVID-19 pandemic [23]. Furthermore, in order to affirm the use of the GT approach, some characteristics had to be met, such as the collection, analysis and coding of data. For example, the collection, analysis and coding of data had to be done simultaneously, non-linearly, so that the comparisons were made continuously [10,24]. Although the methodological and epistemological perspectives are not invariable, it was also needed to still use indispensable processes of theoretical sampling, theoretical saturation, identification of a central category and codifications, which will be further discussed [21]. 

In GT information processing, the construction of memos is extremely important, as it informally portrays the chosen path regarding intuitive ideas, analyses and information debated in the production of knowledge [25]. In this way, these reflective documents help to encourage the investigation and codification of data, development/modification of categories and systematization of thoughts arising from data analysis, contributing to the identification of relations and gaps of knowledge [10,26]. Such memos are present in the study, carried out in the form of informal bulletins, in which the authors explore different concepts, comparisons, exercises and formulas that are were used to guide the construction of the study design and expose relations between the data. At the same time, the dissemination of knowledge about the disease in question is also sought. In general, it is noticeable that the organization in which these methods are configured contributes to the constant articulation of the elements in an iterative and dynamic way [25]. Using the aforementioned GT tools, the research work inaugurates a practical method for the observation of the curve, considering the various influences and biases on its result as a way of indicating parameters for the decision in general. Therefore, this methodology can be simply understood and applied and is not based on modelling with multiple variables in a crisis marked by underreporting, facilitating its replication.

According with the aim of this research, data related to the number of deaths and contaminated people by SARS-CoV-2 in China were initially searched on the platforms of National Health Commission of the People’s Republic of China [27] and Dezan Shira and Associates [28]. Later, the Chinese data were accessed at the Coronavirus Resource Center website of John Hopkins University and Medicine [29]. On the other hand, data from Italy were collected only from John Hopkins University and Medicine [29]. The information was gathered after 13 March 2020. The study period of this work included the active curves, which is the period between the final and initial days but did not discuss the new waves of contamination.

In the case of China, there was a need to reallocate some data accessed on 28 July 2020 which were launched en bloc (large amount in one day). Thus, the abnormal number of contaminated people on 13 February 2020 and deaths detected on 17 April 2020 were redistributed proportionally in the active period of the curve. The non-standard data were subtracted from the trend of progression of the epidemiological curve, leaving 11,102 contaminated people and 1290 deaths to be relocated on the other days already collected, following a weight system in order to not modify the shape of the curve.

In subsequent China database access, on 4 October 2020, the numbers were not displayed in an explicit way, as appeared on 28 July 2020, showing differences in the amount of available data. Thus, it was necessary to reaffirm the previously collected data from the Worldometer info website [30]. The event was checked on the basis of the graphs from contaminated and dead people historic curves, and from the lethality rate of this country.

In the research with GT characteristics, the gathering data resulted in an enormous quantity of those that formed category recognition, which did not correspond to a fixed element. This was perceived as one of the disadvantages of this methodology [31]. However, as the design of the research, based on GT was not previously defined, it was guided by the conceptions built during the investigation of the data providing innovation knowledge [10]. In this sense, during the observation and analysis of the data, the authors tested statistical parameters for the decision and observed the punctual (daily) and accumulated data on deaths and contamination. Besides that, we made an analysis based on a 4-day moving average to eliminate the “weekend effect”, in which data divergence occurred from other days of the week. Apparently, there was no duty on the database management at the weekend, jeopardizing some immediate decisions. In this way, we could take into account the study of Carl Bergstrom [9], which compared the adequate health service capacity or failed healthcare service during the pandemic in an or, on the other hand, failure health management. Both scenarios were exposed as figures with a sinusoidal and symmetrical format that the bases coincided despite having different dimensions. So, we assumed this perfect (symmetric) sinusoidal configuration with only one positive peak between two minimum values for the y axis as an ideal epidemiological curve and using a visual, simple and direct method compared them to the configured (real) curves for China and Italy.

Thus, this ideal curve was mounted on the real curve under development to carry out the approximation and define the parameters of classification and study for decisions. In an attempt to adjust the real curve in this sinusoidal trace, the spots were analyzed. There were points of non-coincidence which represented the differences between the sinusoidal tracing and the COVID-19 curve and consequently a level of risk for underreporting from these gaps. Additionally, it was important to highlight the points of coincidence, matches, which had different meanings in the event’s chronology. For example, start and end dates of the phenomenon considered active or yet events of acceleration in the contamination–fatality curves. As the similarities were confirmed, changes were made in the shape of the ideal curve, preserving the symmetry and proportion of the sides in the sinusoid.

In the analysis of the approximation of the curves, we established rules in the search of matching regions between the two curves and identification of behaviors, also considering the study of punctual (daily) and accumulated data graphics. The correspondence points were a practical way of approaching the curve, called matches (M), with M1 and M3 which was the most important for the analysis of the growth of contamination as shown in Table 1. As 7 Matches approached, there was greater probability of achieving the ideality.

According to the practical observation of various curves of states and countries, an approximation for the greatest success in the treatment of the pandemic indicated a possible and ideal symmetry in the sinusoidal curve. The effectiveness of measures due to the representative of the localities was reflected by the minimum scale of the curve distortion [32]. This search for the sinusoidal curve as an ideal represented the homogeneous effect of dissemination of contamination and the homogeneous effect of isolation against the action of the virus, representing a premise to analyze underreporting and matches. Thus, the match analysis sought for symmetry between the sides, in which the adjust of the theoretical (ideal) curve over the real curve was based on the parameters and positions described as dates and approximations in the shape of the curve (match). The number of cases and fatalities that represented the initial and final impacts of the pandemic were equal in the ideal curve and if underreporting was low, this similarity event was also repeated in the real curve.

To validate the procedure of the adjustments of the different matches, the approximation of the left side—beginning of the curve—and later, of the right side—end of the curve—were considered. On the left side of the curve, the rise of the number of cases could have a continuous shape (surface coincident with the acceleration of the sinusoid), indicating Match. In a practical way, we tried to reduce the differences between the sine wave (ideal curve) and the real curve data to define the ideal peak configuration and by symmetry we defined the shape of the right side of the “mountain”.

In the initial GT-based coding process, when facing the constructed data and graphics, we sought to analyze each part of the data to begin to understand them, what they indicated or could present, inductively producing as many codes as possible (bulletins). This step contributed to directing the research to subsequent data collections that best fit the theory under development [25], undertaking theoretical sampling. This process could be seen in the study in question, as it began with an embracing exploration of the effects of the COVID-19 pandemic on the number of the infected people and deaths in several countries and states. However, by identifying similar patterns in the countries of China and Italy (the first wave), the research was directed to these locations, which led to the beginning of the development of categories.

From the ability to perceive variables and relations through immersion in data (theoretical sensitivity), in the intermediate coding, there was the appearance of a main variable responsible for the behavior of the studied phenomenon [10], which was perceived as the health management against the spread of SARS-CoV-2 in the countries under discussion. In this step, the categories were reviewed and refined by directing them around the central variable [25], to integrate these codes and improve their properties, allowing the emergence of themes [10,24,33], revealed in this research through the parameters. This procedure took place until data saturation was reached, i.e., when new information was not added to the categories even with further analysis [25]. These aspects were described in Table 2 and were used to understand the progression of the ideal and real curve of contaminated and dead people from China and Italy, essential to define the correct moment for the implementation and relaxation of restrictive measures. The indicators, that explain the event, were not pre-existing to the research or were imposed on the data, since they were developed from these, as recommended by the GT [10] and were validated through the application in the real cases in China and Italy.

Finally, the theoretical coding corresponded to the final stage in grounded theory data processing. Unlike the fragmentation present in the initial coding, at this stage, the theoretical codes (parameters) were interconnected to a more embracing, structured and complete theory [25]. This theory established the importance of the developed tools, more discussed in Results and Discussion.

## 3. Results

**Figure 1:**
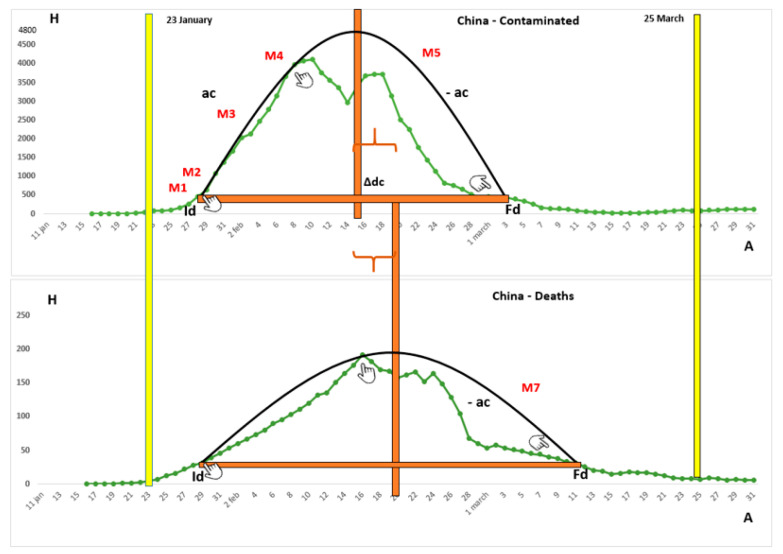
The contaminated people and deaths due to COVID-19 in China. Note. M1, match 1; M2, match 2; M3, match 3; M4, match 4; M5, match 5; M7, match 7; Id, initial date; Fd, final date; ac, acceleration; -ac, deceleration; H, height; A, amplitude; ∆dc, period between the deaths and contaminated peaks of the ideal curve.

**Figure 2:**
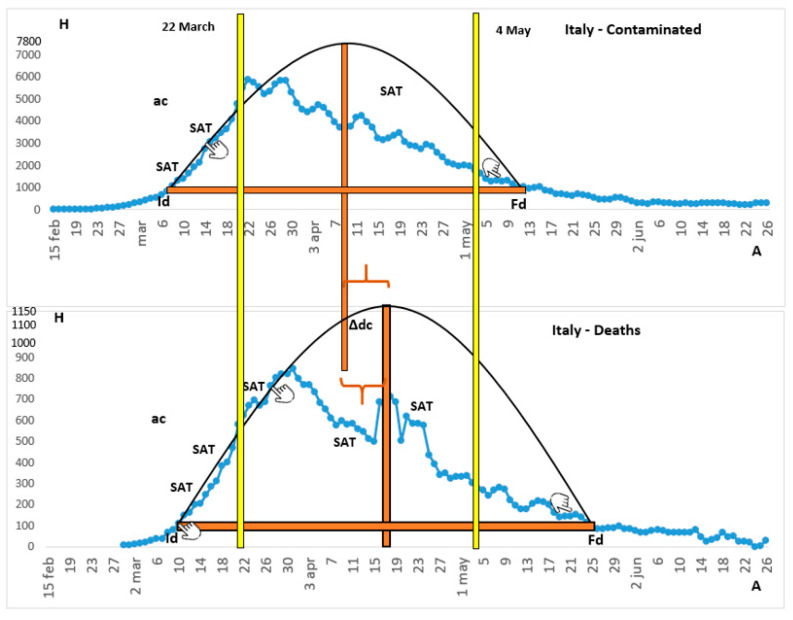
The contaminated people and deaths due to COVID-19 in Italy. Note. Id, initial date; Fd, final date; SAT, saturation point; ac, acceleration; -ac, deceleration; H, height; A, amplitude: ∆dc, period between the deaths and contaminated peaks of the ideal curve.

We assembled and modified the ideal curves according to the matches with the real curves for China and Italy depicted in Figure 1 and Figure 2, respectively. In each figure, the real curve was indicated in green (China) or blue (Italy), in relation to the 4-day moving average of the collected data, and the ideal curve was in black. The yellow bars described the dates of adherence and flexibilization of the widest measures of social restriction in response to the virus. The horizontal bars in red presented the amplitude referring to the active curve, while the red vertical bars symbolized the maximum height reached by the curve in the question, equivalent to the peak.

Among the listed matches, it is possible to state that those between 1 to 5 and 7 were located in figures, while match 6 was the only proximity not seen on the curves. As for the pandemic event, China was the pioneer country in combating SARS-CoV-2 and Italy delayed the initial date of more restrictive measures against the spread of the pathological agent.

When analyzing the real curves of Italy and China, it was visible that, even with the apparent defects, both of them came closer to the ideal curve in the first half of the figure. With reference to the efficiency of notification and data processing, related to the similarity of the real and ideal curves, China reached an index around 80%, while Italy reached 65%.

For the real curves, the amplitude of the death active curve was greater than that of the contaminated people in both countries. The height of the contaminated people curve was much greater than that of dead in these countries, in the proportion of four deaths per 100 contaminated, in China [28] and of 14.3 deaths per 100 contaminated in Italy [29]. In this way, the figures related to death are more flattened than those relating to the contaminated ones, making this notion more accentuated in China than in Italy. Regarding the maximum number of impacts per day of the real and ideal figures, it can be seen that China reached a number equivalent to 4000 and 4800 contaminated and 190 and 200 deaths, while Italy reached 6000 and 7800 contaminated and 800 and 1150 deaths, respectively.

The amplitude of the active contaminated curve in China involved a short period, from 28 January to 3 March, totaling 33 days, while the death curve was greater, since it lasted 38 days, starting on 31 January, stretching to 9 March. Meanwhile, Italy had approximately 67 days between 6 March and 13 May for the contaminated curve, and 80 days for the death curve, which lasted from 8 March to 27 May.

As far as the restrictive measures were concerned, the Italian government adopted, on 22 March, a nationwide quarantine, with the beginning of flexibilization on 4 May. In China, the advent of the lockdown in the regions with the highest number of cases occurred on 23 January and the beginning of the reopening of economic and social activities occurred on 25 March. As for the period between the peaks of the ideal curve of contaminated and deaths, China showed a difference of 6 days and Italy of 9 days.

As for data fluctuations, in China, the curves showed considerable stability. These SATs usually preceded the periods of positive or negative acceleration in the incidence of cases. In Italy, there were marked accelerations at the beginning of the two curves, with a greater angle in the death curve (steeper), while there were no marked decelerations in both the deaths and contaminated data in the country, making the decrease curve for cases less steep. In China, ac was identified only in the contaminated curve and -ac in both graphs, highlighting the death curve (steeper).

It was possible to estimate the lethality of COVID-19 in the two analyzed countries and the result obtained in China was 4% [28] and 14.3% in Italy, respectively [29]. Finally, in relation to risk, it was possible to state that, through the various listed data, an absolute risk value of 4 should be assigned for China and the risk scale between 5 and 6 for Italy. The data presented in this step of the study can be seen in Table 3 and Table 4.

## 4. Discussion

The results showed that the curves of China and Italy approached the ideal curve with an “apparent” beginning and end on the active curve. This curve format was considered as the ideal due to the efficient work of the sampling population, the result of management in mapping the problem and the establishment of isolation programs for the population. Thus, these cases of success (ideal close to the real) in health management indicated: low underreporting; the same order of magnitude of the number of contaminated and dead people; good health management and efficient isolation rate.

The absence of match 6 and smaller approximation in the second half of the curves were related to loosening the data diagnostic and treatment measures carried out by public agencies after the peak of deaths and contamination, the most aggravating moment of the epidemic. It is worth underlining that this behavior of distance from ideality was responsible for the values of efficiency, where China had a better situation and Italy a worse one (85 versus 60%). This problem was linked to the instantaneity of today’s society, in which individuals mobilize under pressure and in emergency situations [34]. So, the discussion on risk aversion and the final stages of critical procedures stood out.

The complexity of interdisciplinary management in connection with the uncertainty of scenarios and data makes it difficult to attribute risk through qualitative and quantitative estimates. However, linking it to the concept of risk, the real economic and social situation, each country has the potential to change the crisis. Thus, the higher the HDI and GDP, the better conditions are to face the consequences of the crisis. In addition, there is still a need for the government perception of the specificities of the population and the condition of the health system.

The risk analysis for critical scenarios indicated that Italy was positioned with average risks between 5 and 6. This assessment was the result of the concentration scenario of COVID-19 cases in the Lombardy region [16], with the amplitude of the active curve of death of 80 days, less deceleration of cases and impacts focused on the elderly population and people with comorbidity [8,35]. On the other hand, China performed contingency quickly and efficiently, concentrating in the city of Wuhan [36], even though it was the first country affected by the new coronavirus [1] with 38 days as the active curve amplitude of deaths, resulting in a risk factor of 4. In both cases, the HDI for successful actions is high and very high, respectively, according to the world ranking 2019 [37].

There was a marked difference in the lethality rate between China (4.3%) and Italy (14%), expressed by the delay in containment measures from the Italian government, the demographics and health situation of the population [35] and the discipline in Chinese oriental culture [15]. It could be also represented by the efficiency of the death curve where, in Italy, this indicator of approximation of the sine wave was 65%, probably showing greater underreporting in relation to China (80%).

China’s quarantine (23 January) and flexibilization (25 March) dates indicated that public agency actions were carried out at the appropriate time. That moment occurred just before Id and after Fd, covering the active curve. In Italy, the isolation date (22 March), which occurred 12 days after the hundredth death, should have started on 27 February, when it reached the 100 contaminated mark as it occurred in China [36]. That fact confirmed a delay of 24 days, as shown in Figure 2.

The management of the database for the pandemic had consequences on the speed of closing and opening of the regions, in which the recognition of saturation points avoided the health planning on the basis of the simulations in the recurrent oscillation of data. In this way, China had no evident saturation, which could be associated with the communication of data blocks informed after the evident impact, indicating that the real shape of the curve could be located in different regions established in the premises of this work when reallocating these atypical data. The major effect of that delay was the communication of the pandemic existence, impacts in high-mobility countries such as China and the speed of the chain reaction [15]. Besides, China underwent the process of adapting knowledge about COVID-19, for example, in respect to the most appropriate tests and characteristic symptoms of the disease, bringing many false positives and negatives [38], in contrast to Italy, where saturations were more evident, due to less rigorous management and the lack of unity of local health programs at the early stages [18].

COVID-19, as a new virus, generated not only a worldwide emergency situation but also led to the increase in the amount of the research on its specific characteristics. In particular, in modeling its curves, it became clear that there was a need to develop models which could incorporate the effect of undetected infected people [39]. Such an approach allowed us to demonstrate the dependence of the impact of COVID-19 on the percentage of the detected cases over the real total infected cases [40]. It was evident that data handling had to chronologically represent the events related to the pandemic. If there was a displacement in the time of contamination or fatality data, or even the non-reporting of these numbers, the decisions on program strategies to combat the crisis and critical analysis to avoid event recurrences were impaired. Thus, from the exploratory method, based on history, we sought to detect under-reporting and redistribute data with abnormal peaks in the curve. In addition, the exploration of China and Italy data was carried out to address motives for the real and ideal curves and contamination and deaths curves presented in the respective formats. Proposing solutions for this non-notification or determining/affirming specific causalities were not part of the scope of this paper.

The dates of acceleration in the rise and fall of the pandemic might be related to the events that occurred during isolation-flexibilization and indicated when and how to build preventive and emergency barriers. For example, if the measures were adopted earlier in Italy, the notable acceleration in the contamination curve from March 6 could have been avoided, reducing the incidence of cases, and decreasing the number of cases could have been more effectively overseen, making the curve down steeper. As they were pioneer countries, it was necessary to mainly evaluate, among these saturation points, the rise of the curve of Italy and the incorrect decisions of public governance regarding the isolation and public health protocols.

The Δdc of 5 days (China) and 8 days (Italy) corresponded to the time between the domestic confirmation of the symptom with mobilization to the hospital and the departure of the body to the morgue. That aspect was directly affected by the ICU occupancy rate in the management of care infrastructures, among the activities of screening, infirmary and intensive care.

The present analysis did not include in-depth details on the groupings between social typologies and sociodemographic, geographic or climatic data in view of the outcome of the pandemic. This work was intended to indicate the main differences between different curves, possible causes and necessary changes in public health activities identified through parameters such as number of days between fatality and contaminated peaks, amplitude and the number of people impacted which could be diminished. The cultural-geopolitical and climatic multi diversity was presented in the shape of the real/ideal curve. Thus, the limits of the improvement or reduction in the impacts and more specifically an indication what happens in crises resulting from unknown dangers in learning also depended on this multidimensionality of several events.

The efficiency in health management in the case of the treatment of epidemics must follow strict protocols and visible indicators that assist the decision on the best security barriers at the time of the demand. The actions against COVID-19 suffered governmental influences and indecisions with the main harmful element being underreporting. The presented parameters are indicators for public policies in crisis management, important for the decision by managers structured on real perceptions of the emerging situation.

## 5. Conclusions

In emerging moments, such as the COVID-19 crisis, new ways of thinking and dealing with situations are required. Therefore, this study provides the relevant theoretical insights and practical applications based on the elements of grounded theory, which is in line with the demands of the current moment: restrictions of resources and deadlines in government programs. The inductive study indicates that the cyclical phenomena of nature follow sine wave formats, bringing a global view of the intensity of crises in society.

The paper discusses the shape of the curve in China and the first wave in Italy, in relation to contamination and fatalities by COVID-19 in order to understand the artificial phenomena (underreporting) and the natural evolution phenomena of the pandemic. Due to the analyzed parameters, it was noticed that, despite having differences in the graphs of cases and deaths, mainly regarding the beginning of the most restrictive measures, China and Italy presented the epidemiological curves close to the ideal with health management and was able to reduce the impacts caused by the pathological agent.

Due to the possibility of underreporting and biased processing of data, it is necessary to understand context in order to make decisions. In this way, this paper demonstrates the importance of researching potentially disastrous or ongoing events, which construction of inductively constructed tools could be the key to damage mitigation. The proposed analysis of the parameters was simple to apply, robust and indicated the regions or issues for discussion in public health as far as the safeguards are applied.

On a practical level, the built categories can assist health managers in optimizing the application resources and defining the best periods for isolation and flexibility of measures focused on mitigating the SARS-CoV-2 pandemic, considering that they provide guidance on the perception of signs of critical levels of the crisis. In this way, the critical sense of health leaders can be developed in efficient and planned crisis management.

In future work, we intend to continue the application and validation of this method by observing these phenomena through the study of the cases of countries that did not perform adequate crisis management, such as Brazil and the United States of America. For this investigation, two parameters will be added: consecutive impact acceleration and new contamination wave.

## Figures and Tables

**Table 1:** Matches and respective descriptions.

Match 1 (M1)	The starting point of coincidence between the real and ideal curves that represented the day on which the contaminated and dead people data were at a relevant value for studies and health planning. It was the beginning of the active curve and it depended on the oscillations in this position.
Match 2 (M2)	The result of the comparison of the number of contaminated people from 50 to 100 and the number of deaths from 1 to 10 with the initial date and the shape of the real–ideal curve, considering that it took a certain period for the impacts to be visualized. This number referred to Pandemic SARS-Cov-2 in relation to the type of event.
Match 3 (M3)	The point of convergence between the curves, which was associated with the confirmation of great growth in the number of people impacted by COVID-19, in an exponential acceleration after the initial period of the curve with saturations in the data.
Match 4 (M4)	Occurring in the periods when the initial curve surface, already configured, coincided with the part of the sinusoidal curve.
Match 5 (M5)	Depending on the future development of the graph or, if it was a historical evaluation, it came from the “descent from the mountain” format. If it was a projection of the future, it was possible to have an idea of what the peak of the curve looked like and, thus, its base moved to establish scenarios according to the efficiency of the isolation rate.
Match 6 (M6)	Deriving from the historical analysis of the curve, representing the comparison and equivalence in the regions of the surface during the decrease in the number of cases or deaths between the ideal curve and the real curve. This point was less usual than M4.
Match 7 (M7)	Related to the downward saturation point and the slowdown of those affected people, corresponding to the final date (Fd) of the active curve under analysis. There might be three Match 7 (Fd1, Fd2 and Fd3) considering the different plans for social flexibility in the countries.

Note. COVID-19, coronavirus disease 2019; Fd, final date; Fd1, final date 1; Fd2, final date 2; Fd3, final date 3; SARS- CoV-2, severe acute respiratory syndrome coronavirus 2.

**Table 2:** Parameters and respective descriptions.

Height (H)	Consequence of the phenomenon in the number of deaths and contaminated people, also related to economic loss, level of poverty and unemployment, and loss of cultural stability. In the worst scenarios, these consequences could lead to higher incidence of behavioral illnesses.
Amplitude (A)	Time referring to the pandemic which could also describe the geographic area of the impacted region if it was related to another disaster such as an earthquake.
Saturation Point (SAT)	Indicative start of the active curve, more visually present in the data on the basis of punctual cases corresponding to an oscillation of values around a local horizontal region. In these cases, the consequences were retained for a period of 4 to 7 days with fluctuating values, present before the change of the contamination curve for an intense acceleration in the contaminated rate. This phenomenon could also occur at the end side of the epidemiological curve, where there is an oscillating repetition to reduce the number of deaths or contaminants, when the restrictive measures were removed.
Initial date (Id)	Initial date of the active curve, depending on saturations in the period of the curve.
Final date (Fd)	Final date(s) of the active curve, resulting from the treatment of risk contamination and available structure for flexibility decisions (Fd1, Fd2, Fd3) where each government must decide about the normalization of activities after risk analysis, respecting the local culture characteristics.
∆dc	Period between the peak of contamination and deaths of the ideal curve (sine wave), indicating strengths and weaknesses in the mobility and occupation of beds in the health system.
Acceleration (ac)	Sharp increase in daily velocity (ac), from one day or period to another or sharp deceleration (-ac) in the number of contaminated or dead people. It represented the change in the angle in the rise and fall of the “mountain”.
Lethality (LET)	Ratio between the number of deaths and the total number of contaminated patients with the new coronavirus. We used accumulated lethality, but it could be used to correct public health decisions, when the parameter was the moving average of punctual lethality, respecting ∆dc.
Efficiency (EFFI)	Percentage of the efficiency of the curve, which qualified health management in terms of measures related to data notification and treatment.
Event	Specific characteristic, natural or social, in the period of acceleration and peak, corresponding to the local peculiarity that affected the fight against COVID-19.
Risk	Qualitative aspect based on demography, human development index (HDI), gross domestic product (GDP) and hospital practices. The risk management of the object of the study was evaluated and had a graduation from 1 to 10, with 1 symbolizing the minimum risk of contamination and 10, the maximum risk.

Note. Fd1, final date 1; Fd2, final date 2; Fd3, final date 3; ac, acceleration; -ac, deceleration; COVID-19, coronavirus disease 2019.

**Table 3:** Results of the parameters. Part 1.

Country	H Real		A Real		SAT		Id		Fd	
	C	D	C	D	C	D	C	D	C	D
China	4000	200	33	38			28 Jan	31 Jan	3 Mar	9 Mar
Italy	6000	800	67	80	Moderate: Both halves of the graph	Moderate: Both halves of the graph	6 Mar	8 Mar	13 May	27 May

Note. H, height; A, amplitude; SAT, saturation point; Id, initial date; Fd, final date; C, contaminated; D, deaths; Jan, January; Mar, March; Feb, February.

**Table 4:** Results of the parameters. Part 2.

Country	ac				∆dc	LET (%)	Risk	EFFI (%)	Event
	C		D						
	+	−	+	−					
China	29 Jan to 7 Feb	19 to 25 Feb		24 to 28 Feb	5	4	4	80	Pioneer
Italy	9 to 19 Mar		11 to 21 Mar		8	14.3	5–6	65	Delayed action

Note. C, contaminated; D, deaths; ac, acceleration; ∆dc, period between the deaths and contaminated peaks of the ideal curve; LET, lethality; EFFI, efficiency; Jan, January; Mar, March; Feb, February.

## Data Availability

The data presented in this study are openly available in FigShare at 10.6084/m9.figshare.13562348.
